# Human α-Defensin 5_1–9_ and Human β-Defensin 2 Improve Metabolic Parameters and Gut Barrier Function in Mice Fed a Western-Style Diet

**DOI:** 10.3390/ijms241813878

**Published:** 2023-09-09

**Authors:** Louisa Filipe Rosa, Andreas Rings, Iris Stolzer, Louis Koeninger, Jan Wehkamp, Julia Beisner, Claudia Günther, Peter Nordkild, Benjamin A. H. Jensen, Stephan C. Bischoff

**Affiliations:** 1 Institute of Nutritional Medicine, University of Hohenheim, Fruwirthstr. 12, 70599 Stuttgart, Germany; 2 Department of Medicine 1, Universitätsklinikum Erlangen, Friedrich-Alexander-Universität Erlangen-Nürnberg, 91054 Erlangen, Germany; 3 Deutsches Zentrum Immuntherapie (DZI), Universitätsklinikum Erlangen, 91054 Erlangen, Germany; 4 Department of Internal Medicine I, University Hospital Tübingen, 72016 Tübingen, Germany; 5 Defensin Therapeutics, 2200 Copenhagen, Denmark; 6 Department of Biomedical Sciences, Faculty of Health and Medical Sciences, University of Copenhagen, 1353 Copenhagen, Denmark

**Keywords:** HD5 fragments, hBD2, host defense peptides, antimicrobial peptides, gut barrier, obesity, NAFLD, glucose metabolism

## Abstract

Obesity and metabolic comorbidities are associated with gut permeability. While high-fructose and Western-style diet (WSD) disrupt intestinal barrier function, oral administration of human α-defensin 5 (HD5) and β-defensin 2 (hBD2) is believed to improve intestinal integrity and metabolic disorders. Eighty-four male C57BL/6J mice were fed a WSD or a control diet (CD) ± fructose (F) for 18 weeks. In week 13, mice were randomly divided into three intervention groups, receiving defensin fragment HD5_1–9_, full-length hBD2, or bovine serum albumin (BSA)-control for six weeks. Subsequently, parameters of hepatic steatosis, glucose metabolism, and gut barrier function were assessed. WSDF increased body weight and hepatic steatosis (*p* < 0.01) compared to CD-fed mice, whereas peptide intervention decreased liver fat (*p* < 0.05) and number of hepatic lipid droplets (*p* < 0.01) compared to BSA-control. In addition, both peptides attenuated glucose intolerance by reducing blood glucose curves in WSDF-fed mice. Evaluation of gut barrier function revealed that HD5_1–9_ and hBD2 improve intestinal integrity by upregulating tight junction and mucin expression. Moreover, peptide treatment restored ileal host defense peptides (HDP) expression, likely by modulating the Wnt, Myd88, p38, and Jak/STAT pathways. These findings strongly suggest that α- and β-defensin treatment improve hepatic steatosis, glucose metabolism, and gut barrier function.

## 1. Introduction

Gut barrier impairment has been associated with a number of chronic diseases, including obesity and associated metabolic diseases, such as type 2 diabetes [1], nonalcoholic fatty liver disease (NAFLD), and nonalcoholic steatohepatitis (NASH) [2,3]. Bacterial translocation, enabled by deteriorated gut barrier function, in human obesity is aggravated in patients with type 2 diabetes compared to their weight-matched, normoglycemic counterparts [4]. Still, the mechanisms, by which gut barrier function is impaired and how this might affect obesity-related comorbidities are largely unclear [5]. We and others have shown that high fructose intake, along with high-fat and high-sugar diet, the so called Western-style diet (WSD), increase permeability of intestinal barrier in rodents [6,7,8] and likely also in humans [9,10,11]. Gut barrier impairments lead to increased translocation of bacterial endotoxins and probably even whole bacteria from the gut into the portal vein system and liver, promoting liver inflammation and liver steatosis [12,13,14]. Low-grade inflammation, which is a direct consequence of gut barrier impairment, has been recognized as a relevant risk factor for development of metabolic diseases [3,15]. While the relation between gut barrier impairment and metabolic diseases has become clear during recent years, attempts to restore gut barrier function are limited and mostly speculative. Both exogenous factors, including dietary factors such as fiber [16,17] and probiotics [18], as well as endogenous factors, such as mucus or defensins, are conceptual treatment options. Defensins (also termed cryptidins in mice) are an important component in the intestinal barrier’s first line of defense [19,20]. Defensins are small, cationic peptides with a characteristic folded β-structure, stabilized by three intramolecular disulfide bonds. Accordingly, they are classified into alpha-, beta-, and delta-defensins [20,21], as disulfide bonds are formed in α-defensins at position 1–6, 2–4, and 3–5, whereas β-defensins contain three disulfide bonds at 1–5, 2–4, and 3–6 [22,23]. While human α-defensins 5 and 6 are mainly expressed by Paneth cells in the small intestine, β-defensins are produced at epithelial surfaces and in the colon [20], but also in other organs such as liver [24]. HD5 and HD6 are the predominantly expressed α-defensins in the small intestine, which also exhibit high antimicrobial activity against pathogenic bacteria in the intestinal lumen [25]. Upon proteolytic degradation in the reduced small intestinal environment, HD5 is cleaved to numerous fragments with biological activity, of which especially fragment HD5_1–9_, consisting of nine amino acids (ATCYCRTGR), showed good antimicrobial activity in vitro [26].

Human studies revealed that the onset of obesity is associated with decreased intestinal α-defensin mRNA expression [27]. Moreover, reduced intestinal α-defensin expression disturbed the composition of intestinal microbiota and led to bacterial overgrowth in the gut, and decreased intestinal mucosal integrity [28,29,30]. Consistently, patients with (de-) compensated liver cirrhosis showed decreased intestinal expression of human α-defensins 5 (HD5) and 6 (HD6) [31]. In mice, oral administration of human HD5 improved diet-induced hepatic steatosis and dyslipidemia, and increased ileal expression of the murine analog DEFA5 [32,33,34]. In further mouse studies, overexpression of full HD5 peptide increased ileal bacterial diversity and treatment with HD5_1–9_ fragment enhanced the abundance of *Akkermansia muciniphila* [26,35,36]. Further, it has been demonstrated that treatment of mice with a modified HD5_1–9_ fragment, termed D3, protected against obesity by suppressing appetite and regulating gut microbiota [37]. Therapeutic effects on barrier function were also shown for β-defensins in mice, as systemic administration of human β-defensin 2 (hBD2) reduced weight loss and disease activity index in three different colitis models in mice [38]. hBD2 is a low molecular weight peptide consisting of 64 amino acids with positive net charges in its primary structure, providing antimicrobial activity by binding to negatively charged molecules on the surface of bacteria [39]. In vitro, hBD-2 has been found to reduce proinflammatory cytokines, such as interleukin 1-β (IL1-β) and TNF-α (TNF) in LPS-treated human peripheral blood mononuclear cells (PBMCs) [38]. Furthermore, oral administration of hBD-2 reduced both alcohol-induced liver injury and graft-versus-host disease, in numerous mouse strains, by improving barrier function and thereby modulating host–microbe interactions and immunoreactivity [40,41].

While high-fructose and Western-style diet has been associated with intestinal barrier dysfunctions, and in particular impaired expression of host defense peptides (HDPs), oral administration of full-length hBD2 and HD5 has been found to improve intestinal barrier integrity and obesity-related comorbidities, respectively. In the present study, we examined the effects of oral administration of HD5 peptide fragment HD5_1–9_ and full peptide β-defensin 2 (hBD-2) on intestinal barrier function and metabolic diseases in male C57BL/6J mice fed a high-fructose or Western-style diet.

## 2. Results

### 2.1. Oral HD5_1–9_ and hBD2 Administration Partially Improve WSD±F Induced Deterioration in Body Weight, Mesenteric Fat Tissue, and Hepatic Steatosis

**Figure 1:**
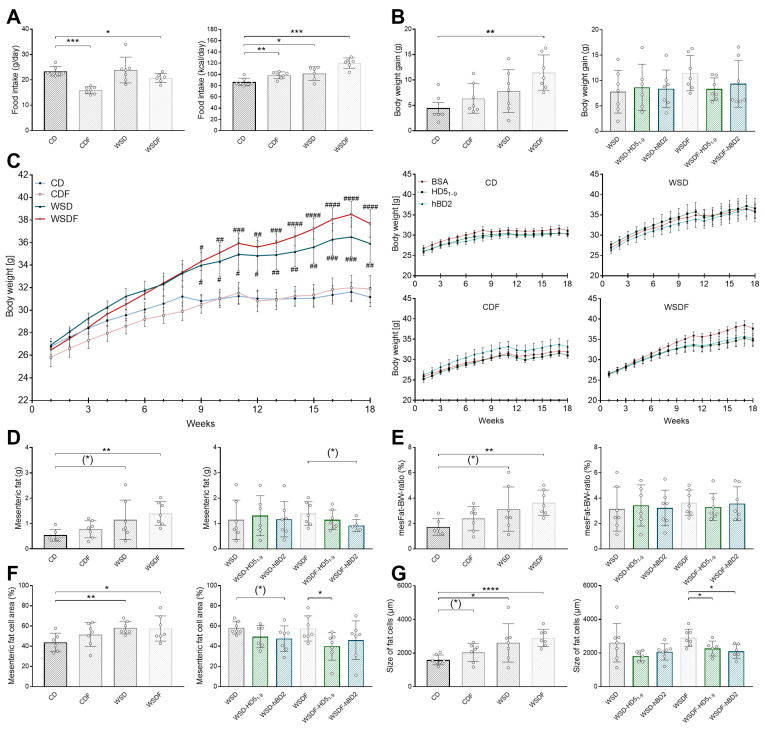
WSD±F-fed mice exhibited increased body weight gain and mesenteric fat accumulation, which was partially reduced by defensin administration (food intake in g/day and energy intake in kcal/day during the 18-week feeding period) (**A**). Weight gain in g (**B**) and weight development of mice for 18 weeks of diet and peptide intervention (**C**). Mesenteric fat in g (**D**), mesenteric fat-to-body weight (mesFat-BW) ratio (**E**), mesenteric fat cell area in % (**F**), and fat cell size in µm (**G**) after 18-week feeding period are shown. Data are presented as means +/− standard error of the mean (*n* = 6–7; graphically indicated by ° for one sample each). Statistical analysis was performed using the Kruskal–Wallis test with Dunn’s test (**A**,**B**,**D**,**E**), or by one-way ANOVA with Dunnett’s post-test (**E**,**F**), or by two-way ANOVA with Tukey’s multiple comparisons test (**C**). Significant differences are indicated: * *p*-value < 0.05; ** *p*-value < 0.01; *** *p*-value < 0.001; **** *p*-value < 0.0001. (*) *p*-values ranging between > 0.05 and < 0.1 were considered to indicate a trend. # indicates differences relative to WSDF. # *p*-value < 0.05; ## *p*-value < 0.01; ### *p*-value < 0.001; #### *p*-value < 0.0001 Abbreviations: CD, control diet; F, fructose; HD5_1–9_, human α-defensin 5 peptide fragment 1–9; hBD2, human β-defensin 2; mesFat-BW ratio, mesenteric fat-to-body weight ratio; WSD, Western-style diet.

Eighteen-week feeding of high-fructose solution reduced total food intake in both CD- (*p* < 0.001) and WSD-fed mice (*p* < 0.05) compared to their nonfructose-fed counterparts. However, despite decreased food intake, energy intake was increased in mice fed either CDF (*p* < 0.01) or WSDF (*p* < 0.001). Mice fed WSD also consumed more energy than the CD-fed control group (*p* < 0.05) (Figure 1A). While body weight gain in g was only increased in WSDF mice (*p* < 0.01) (Figure 1B), weight development during the 18-week feeding period increased significantly from week 9 onwards in WSD±F-fed mice (*p* < 0.05) compared to mice receiving CD (Figure 1C). Moreover, weight development continued to increase up to week 18 for both WSD (*p* < 0.01) and WSDF (*p* < 0.0001) (Figure 1C). In addition, fat tissue measurements revealed that mesenteric fat in g and mesenteric fat-to-body weight (mesFat-BW) ratio were only significantly increased in WSDF-fed (*p* < 0.01), whereas both mesenteric fat weight (*p* = 0.094) and mesFat-BW ratio (*p* = 0.088) only tended to be increased in WSD-fed mice (Figure 1D,E). These results point toward the fact that high-fat and high-sugar diet increased body weight especially by accumulation of mesenteric fat tissue, whereas high consumption of fructose had no effect on body weight and fat tissue (Figure 1B,D).

While therapeutic intervention with either HD5_1–9_ or hBD2 had no effect on body weight gain (Figure 1B) or body weight development (Figure 1C), 6 weeks of hBD2 administration tended to decrease mesenteric fat (*p* = 0.065) (Figure 1D) but not mesFat-BW ratio in WSDF-fed mice (*p* = 0.9) (Figure 1E). Histological analysis of mesenteric fat tissue revealed that CD-fed mice exhibited a mesenteric fat cell area of 44%, whereas 18-week feeding of WSD (*p* < 0.01), and WSDF (*p* < 0.05) increased mesenteric fat cell area in mice (Figure 1F) (Figure S1). Moreover, mesenteric fat cell size was only significantly increased in WSDF-fed mice (*p* < 0.001), although both CDF (*p* = 0.094) and WSD feeding (*p* = 0.072) showed similar tendencies (Figure 1G). In comparison, 6-week treatment with HD5_1–9_ decreased mesenteric fat cell area in WSDF-fed mice (*p* < 0.05) and tended to decrease fat cell size (*p* = 0.084). Similarly, WSDF-fed mice treated with hBD2 exhibited a reduced fat cell size (*p* < 0.05) but not fat cell area (*p* = 0.2) (Figure 1F,G).

**Figure 2:**
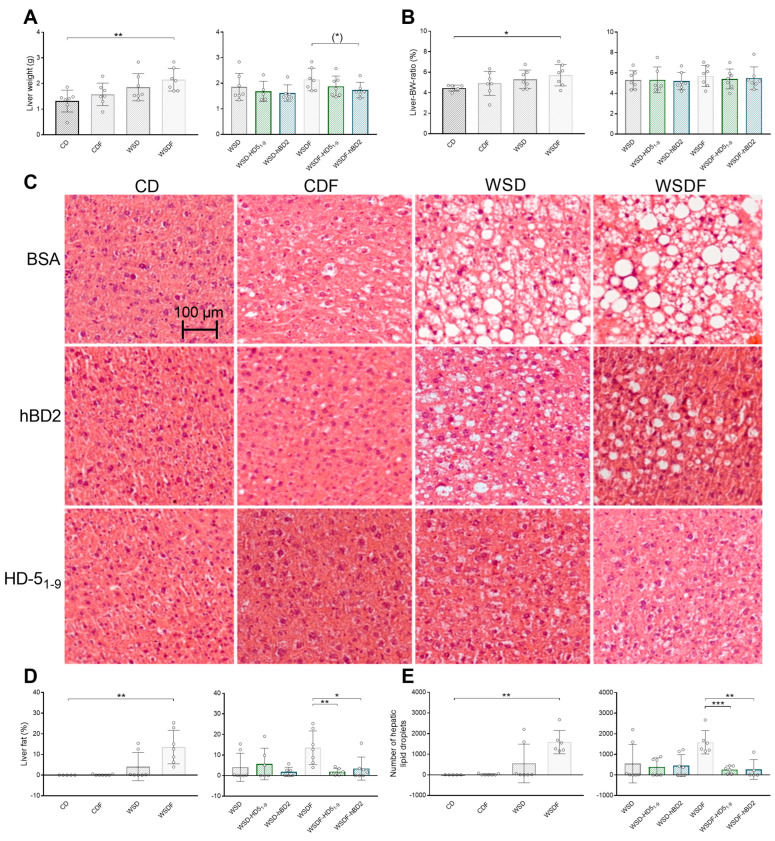
Oral administration of antimicrobial peptides reduced hepatic steatosis in mice fed WSDF. Liver weight in g (**A**) and liver-to-bodyweight (Liver-BW) ratio (**B**) after an 18-week feeding period are shown. Representative images of HE staining of hepatic tissue. Scale bar: 50 µm (**C**). Liver fat in % (**D**) and total number of hepatic lipid droplets (**E**) are shown. Data are presented as means +/− standard error of the mean (*n* = 6–7; graphically indicated by ° for one sample each). Statistical analysis was performed by one-way ANOVA with Dunnett´s post-test (**A**,**B**) or by the Kruskal–Wallis test with Dunn’s test (**C**,**E**). Significant differences are indicated: * *p*-value < 0.05; ** *p*-value < 0.01; *** *p*-value < 0.001. (*) *p*-values ranging between > 0.05 and < 0.1 were considered to indicate a trend. Abbreviations: CD, control diet; F, fructose; HD5, human α-defensin 5; hBD2, human β-defensin 2; Liver-BW ratio, Liver-to-body weight ratio; WSD, Western-style diet.

Next, we analyzed hepatic steatosis. WSDF feeding increased liver weight (*p* < 0.01) (Figure 2A) and liver-to-body weight (Liver-BW) ratio (*p* < 0.05) (Figure 2B). Moreover, a 6-week administration of hBD2 (*p* = 0.081) but not HD5_1–9_ (*p* = 0.3) tended to reduce liver weight (Figure 2A), whereas Liver-BW ratio was unaffected in WSDF-fed mice (Figure 2B).

Concordantly, histological quantification demonstrated that WSDF increased liver fat (*p* < 0.01) and the number of hepatic lipid droplets (*p* < 0.001) (Figure 2C–E). Interestingly, HD5_1–9_ (*p* < 0.01) or hBD2 administration decreased (*p* < 0.05) liver fat (Figure 2C,D), and both peptide interventions reduced the number of hepatic lipid droplets in these mice (*p* < 0.01) (Figure 2E).

As high fructose intake and WSD have been associated with bacterial translocation [3,42], we next measured endotoxin concentration in portal vein plasma. However, neither diets nor treatment with HD5_1–9_ or hBD2 had any effect on plasma endotoxin concentrations in mice (Figure S2). Nevertheless, liver weight and fat analysis demonstrated that WSDF resulted in hepatic steatosis, whereby HD5_1–9_ or hBD2 treatment reduced hepatic fat and number of hepatic lipid droplets. These findings suggest that HD5_1–9_ or hBD2 attenuate hepatic steatosis and thus could be useful for the treatment and prevention of metabolic liver diseases such as NAFLD.

### 2.2. Oral Administration of HD5_1–9_ and hBD2 Attenuate Glucose Intolerance in Mice Fed a WSDF

**Figure 3:**
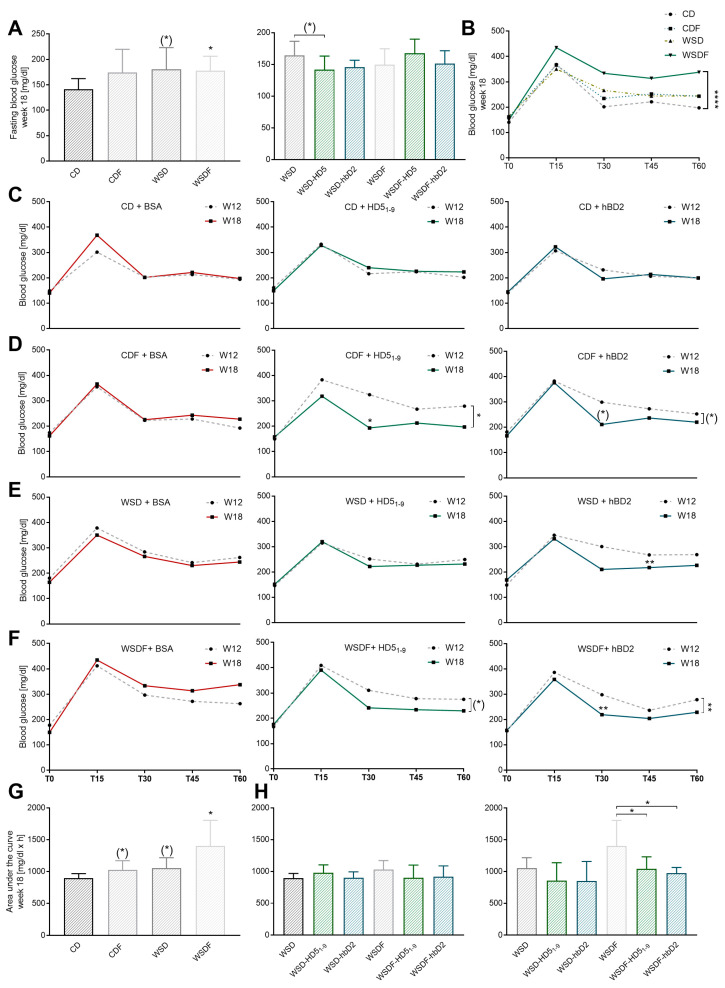
HD5_1–9_ and hBD2 attenuated glucose tolerance in mice fed high-fructose diets. Fasting blood glucose in mg/dl (**A**) and changes in blood glucose from administration of glucose to the 15, 30, 45, and 60 min mark (**B**) after the 18-week feeding period are shown. Changes in blood glucose after BSA or HD5_1–9_ or hBD2 treatment in mice fed a CD (**C**), CDF (**D**), WSD €, or WSDF (**F**) for 18 weeks. Area under the curve (AUC) in mg/dl x h after the 18-week feeding period (**G**) and after peptide treatment (**H**) are shown. Data are presented as means +/− standard error of the mean (*n* = 6–7; graphically indicated by ° for one sample each). Statistical analysis was performed by one-way ANOVA with Dunnett´s post-test (**A**) or by two-way ANOVA with Tukey’s multiple comparisons test (**B**–**F**). For nonparametric data, the Kruskal–Wallis test with Dunn’s test was performed (**G**,**H**). Significant differences are indicated: * *p*-value < 0.05; ** *p*-value < 0.01; **** *p*-value < 0.0001. (*) *p*-values ranging between > 0.05 and < 0.1 were considered to indicate a trend. Abbreviations: AUC, area under the curve; CD, control diet; F, fructose; HD5, human α-defensin 5; hBD2, human β-defensin 2; WSD, Western-style diet.

Assessment of oral glucose tolerance test (oGTT) revealed that 18-week feeding of WSD (*p* = 0.067) or WSDF (*p* < 0.05) increased fasting blood glucose levels in mice (Figure 3A). Similarly, 18-week feeding of WSDF increased blood glucose curves after administration of a glucose bolus to 15, 30, 45, and 60 min time point post-glucose challenge (*p* < 0.0001) (Figure 3B). Moreover, 6-week intervention with HD5_1–9_ reduced fasting blood glucose in WSD-fed mice by trend (*p* = 0.087), whereas hBD2 had no effect (*p* = 0.9) (Figure 3A). To evaluate the effects of HD5_1–9_ and hBD2 on glucose tolerance, we examined changes in blood glucose following a glucose bolus before (week 12) and after (week 18) treatment for each diet (Figure 3C–F). Administration of BSA-control did not change blood glucose curves in either diet (Figure 3C–F). Similarly, there were no changes in healthy CD-fed mice receiving HD5_1–9_ or hBD2 (Figure 3C). However, in mice receiving a high-fructose solution, HD5_1–9_ improved glucose tolerance, indicated by a significant reduction at time T30 (*p* < 0.05) and T60 (*p* < 0.05), and supported by a similar trend in WSDF-fed mice (*p* = 0.065) (Figure 3F). Comparable trajectories were observed for hBD2, with a seemingly improved blood glucose curve in CDF-fed mice (*p* = 0.066) and a significantly improved glucose tolerance in WSDF-fed mice (*p* < 0.01) (Figure 3D,F). Additionally, hBD2 but not HD5_1–9_ administration enhanced glucose tolerance in WSD-fed mice (T45: *p* < 0.01) (Figure 3E). Next, we determined the area under the curve (AUC) (Figure 3G,H). CDF by trend (*p* = 0.086), WSD by trend (*p* = 0.07), and WSDF-fed mice (*p* < 0.01) exhibited increased AUC compared to CD-controls (Figure 3G). Oral administration of HD5_1–9_, and hBD2, did not change AUC in CDF- and WSD-fed mice, but both treatments reduced AUC in WSDF-fed mice (*p* < 0.05). Collectively, our results indicate that both HD5_1–9_ and hBD2 improve diet-induced disorders in glucose metabolism.

### 2.3. HD5_1–9_ and hBD2 Treatment Improves Intestinal Barrier Function by Regulating Tight Junction and Mucin Expression

As obesity and metabolic diseases such as NAFLD have been associated with impaired intestinal barrier [43], we next analyzed parameters of gut barrier function. Histological evaluations of the ileum and colon revealed that neither diets nor HD5_1–9_ or hBD2 treatment had any effect on intestinal morphology (Figure S3). A Lac/Man permeability assay revealed that 6 weeks of HD5_1–9_ administration reduced the Lac/Man ratio in both WSD- (*p* < 0.01) and WSDF-fed mice (*p* < 0.05). Similar patterns were observed for hBD2 in WSD-fed (*p* < 0.05) but not in WSDF-fed mice (*p* = 0.6) (Figure S4).

**Figure 4:**
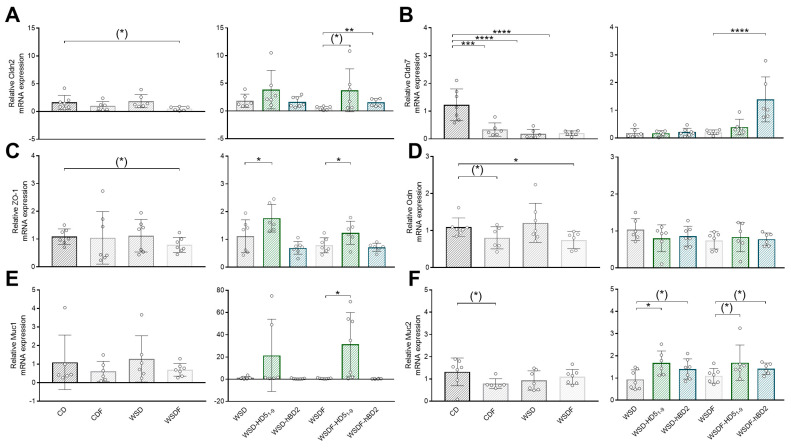
Peptide treatment regulated gut barrier function by inducing gene expression of tight junction proteins and mucins. Ileal mRNA expression levels of cldn2 (**A**), cldn7 (**B**), ZO-1 (**C**), ocln (**D**), Muc1 (**E**), Muc2 (**F**) after the 18-week feeding period are shown. Data are presented as means +/− standard error of the mean (*n* = 6–7; graphically indicated by ° for one sample each). Statistical analysis was performed by one-way ANOVA with Dunnett´s post-test (**A**) or by the Kruskal–Wallis test with Dunn’s test (**B**–**F**). Significant differences are indicated: * *p*-value < 0.05; ** *p*-value < 0.01; *** *p*-value < 0.001; **** *p*-value < 0.0001. (*) *p*-values ranging between > 0.05 and < 0.1 were considered to indicate a trend. Abbreviations: cldn, claudin; Muc, mucin; ocln, occludin; ZO-1, zonula occludens 1. For other abbreviations, see Figure 1.

Since TJ proteins regulate paracellular transport and thus have a crucial role for the intestinal barrier function [44], we next measured TJ gene expression in the ileum and colon of mice. PCR analysis revealed that WSDF tended to decrease ileal gene expression of claudin (*cldn*) 2 (*p* = 0.056), whereas expression was upregulated by a trend in HD5_1–9_-treated mice (*p* = 0.069). Additionally, hBD2 induced ileal *cldn2* mRNA expression (*p* < 0.01) (Figure 4A). In the colon, 18-week feeding of CDF reduced cldn2 expression (*p* < 0.05), whereas neither HD5_1–9_ nor hBD2 had any effect on *cldn2* gene expression (Figure S5). Ileal *cldn7* expression was reduced by CDF (*p* < 0.001), WSD (*p* < 0.0001), and WSDF (*p* < 0.0001). Importantly, hBD2 treatment of WSDF-fed mice restored this marked decrease in ileal *cldn7* mRNA expression (*p* < 0.0001) (Figure 4B). Moreover, the HD5 peptide fragment and hBD2 prevented WSDF-induced reductions in *cldn7* gene expression in the colon of mice (*p* < 0.05) (Figure S5). Comparable, ileal expression of zonula occludens (*ZO-1*) tended to be downregulated by WSDF feeding (*p* = 0.062), whereas HD5_1–9_ but not hBD2 upregulated *ZO-1* expression in the ileum of WSD±F-fed mice (*p* < 0.05) (Figure 4C). While *ZO-1* expression in the colon remained unchanged by dietary intervention, 6-week treatment with HD5_1–9_ exhibited a similar tendency as in the ileum (*p* = 0.096) (Figure S5). Although ileal occludin (*ocln*) mRNA expression was reduced in CDF-fed mice by trend (*p* = 0.083), and in WSDF-fed mice (*p* < 0.05), neither HD5_1–9_ nor hBD2 treatment had any effect (Figure 4D). However, despite the apparent preference for ileal rather than colon restoration, as outlined above, PCR analysis of colon samples revealed enhanced *ocln* mRNA expression levels in WSD- (*p* < 0.001) and WSDF-fed mice (*p* < 0.05) treated with HD5_1–9_, whereas hBD2 administration had no effect (Figure S5).

Another component of the intestinal barrier function is the mucus layer. Measurements in the ileum revealed that neither mucin (*Muc*) 1 nor *Muc2* were affected by WSD±F, whereas 18-week feeding of CDF tended to reduced ileal *Muc2* expression (*p* = 0.074) (Figure 4E,F). Moreover, measurements in the colon revealed a reduction in *Muc1* by trend (*p* = 0.061), when mice were fed a WSDF (Figure S5). CDF-fed mice exhibited a similar tendency to reduced colonic *Muc2* expression (*p* = 0.073) (Figure S5). We were able to show that administration of HD5_1–9_ induced ileal *Muc1* gene expression in WSDF-fed mice (*p* < 0.05) (Figure 4E), and ileal *Muc2* mRNA expression in WSD- (*p* < 0.05) and WSDF-fed mice by trend (*p* = 0.098) (Figure 4F). We observed similar trajectories for hBD2-treated mice with an apparent upregulation of ileal *Muc2* expression in WSD- (*p* = 0.07) and WSDF-fed mice (*p* = 0.08). In contrast, peptide treatment had no effect on *Muc1* or *Muc2* expression in the colon (Figure S5).

In summary, our results imply that both HD5_1–9_ and hBD2 enhance intestinal barrier function and integrity by modulating different gut barrier components.

### 2.4. HD5_1–9_ and hBD2 Administration Restores High-Fructose- and WSD-Induced Impairments in Host Defense Peptide Secretion

**Figure 5:**
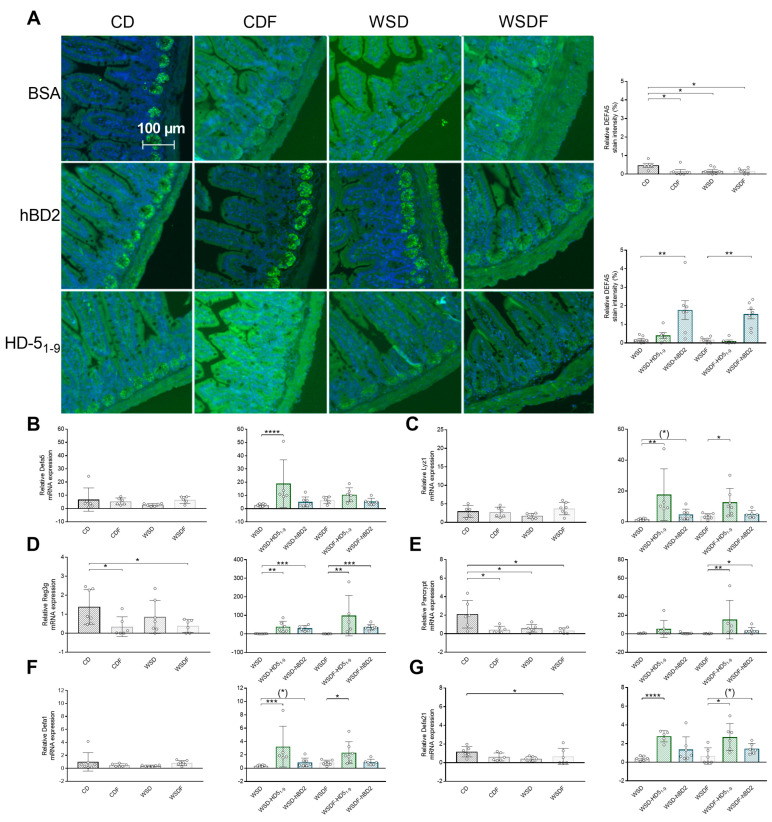
HD5_1-9_ and hBD2 administration attenuated antimicrobial peptides in mice fed high-fat, high-sugar, and high-fructose diets. Representative images show DEFA5 expression (green) and cell nuclei detected by DAPI (blue). Scale bar: 50 µm. Quantification of relative DEFA5 fluorescence intensity in percent (**A**). mRNA expression levels of Defa5 (**B**), Lyz1 (**C**), Reg3g (**D**), pan-cryptidin €, Defa1 (**F**), and Defa21 (**G**) after the 18-week feeding period are shown. Data are presented as means +/− standard error of the mean (*n* = 6–7; graphically indicated by ° for one sample each). Statistical analysis was performed by the Kruskal–Wallis test with Dunn’s test (**A**,**B**,**E**,**F**) or by one-way ANOVA with Dunnett´s post-test (**C**,**D**,**G**). Significant differences are indicated: * *p*-value < 0.05; ** *p*-value < 0.01; *** *p*-value < 0.001; **** *p*-value < 0.0001. *p*-value < 0.0001. (*) *p*-values ranging between > 0.05 and < 0.1 were considered to indicate a trend. Abbreviations: DEFA5, α-defensin 5; Lyz1, lysozyme; Reg3g, regenerating islet-derived protein 3 gamma; Defa1, cryptidin 1; Defa21, cryptidin 4; Pancrypt, pancryptidin. For other abbreviations, see Figure 1.

High-fructose diets have been shown to compromise Paneth cell function [45], so we next examined small intestinal HDPs. Assessment of ileal immunohistochemically DEFA5 staining, which is the murine analogue to HD5, revealed that CD-fed mice expressed DEFA5 protein at the base of crypts in Paneth cell granules (Figure 5A), whereas DEFA5 protein levels were almost absent in mice fed a CDF, WSD, or WSDF (*p* < 0.05) (Figure 5A). Interestingly, hBD2 but not HD5_1–9_ administration increased DEFA5 protein signal in WSD- and WSDF-fed mice (*p* < 0.001) (Figure 5A). In contrast to protein abundance, PCR measurements revealed that neither CDF nor WSD±F had any effect on ileal *Defa5* mRNA expression. Despite the lack of dietary impact on mRNA expression, oral administration of HD5_1–9_ induced *Defa5* expression in WSD-fed mice (*p* < 0.0001), a pattern that was not recapitulated in hBD2-treated mice (*p* = 0.2) (Figure 5B).

Since lysozyme and Reg3γ have been shown to improve intestinal barrier function [46,47], we evaluated gene expression of these HDPs. While diets had no effects on lysozyme expression (Figure 5C), feeding CDF (*p* < 0.05) or WSDF (*p* < 0.05) reduced Reg3γ mRNA expression (Figure 5D). Pointing toward enhanced barrier function in the defensin-administered mice, WSD-fed mice treated with HD5_1–9_ increased lysozyme (*p* < 0.01) and Reg3γ (*p* < 0.01), supported by a similar trend in hBD2-treated mice for lysozyme expression (*p* = 0.065) and Reg3γ (*p* < 0.001) (Figure 5C,D). Complementarily, WSDF-fed mice, treated with HD5_1–9_ (*p* < 0.01) or hBD2 (*p* < 0.001), exhibited increased Reg3γ mRNA levels, whereas lysozyme expression was induced only in WSDF-fed mice receiving the HD5 peptide fragment (*p* < 0.05) but not hBD2 (Figure 5C,D).

Assessment of total cryptidin by pan-cryptidin assay revealed that both high fructose consumption and WSD decreased pan-cryptidin gene expression (*p* < 0.05) (Figure 5E). In addition, oral administration of HD5_1–9_ (*p* < 0.01) or hBD2 (*p* < 0.05) induced ileal pan-cryptidin gene expression in WSDF-fed mice, (Figure 5E). Quantification of ileal cryptidin 1 and 4 gene expression revealed that only WSDF decreased the expression of cryptidin 4 (*p* = 0.05), whereas the other diets had no effect (Figure 5F,G). While treatment of WSD-fed mice with hBD2 only tended to induce cryptidin 1 mRNA expression (*p* = 0.083), HD5_1–9_ promoted significant induction of cryptidin 1 (*p* < 0.001) and 4 (*p* < 0.0001) (Figure 5F,G). Moreover, WSDF-fed mice receiving hBD2 showed increased cryptidin 4 gene expression by trend (*p* = 0.093), while HD5_1–9_ treatment enhanced the expression of both cryptidin 1 (*p* < 0.05) and 4 (*p* < 0.05) in these mice (Figure 5F,G).

The Wnt signaling pathway is an important regulator of intestinal HDP defense. We therefore hypothesized that peptide treatment modulates Wnt signaling activity. Quantification in the ileum found that none of the diets affected Wnt signaling pathway activity (Table 1). Still, we were able to show that HD5_1–9_ but not hBD2 modulated the Wnt signaling pathway, by inducing *Wnt3* (*p* < 0.01), *Wnt5a* (*p* < 0.05), and *Wnt9a* (*p* < 0.01) gene expression (Table 1). While Wnt signal transmitting receptor *LRP6* was not affected, HD5_1–9_ induced intracellular transcription factor *Tcf1* (*p* < 0.01) (Table 1).

Next, we analyzed *Mmp7* gene expression, as the enzyme is required for proteolytic activation of antimicrobial peptides. However, PCR measurements revealed that neither diets nor peptide treatments had any effect on *Mmp7* mRNA levels (Table 1). In conclusion, these data point toward the fact that peptide treatment ameliorated small intestinal gut barrier function by regulating HDP defense and that the Wnt signaling pathway might be involved in HD5_1–9_-mediated effects.

### 2.5. HD5_1–9_ and hBD2 Modulate HDP Defense In Vitro through the Myd88, p38, and Jak/STAT Signaling Pathways

There is evidence that HDPs provide several immunomodulatory effects through the TLR4/Myd88, p38/MAPK, and Jak/STAT pathways [48,49,50]. Therefore, we next used an ex vivo small intestinal organoid cell model to evaluate whether these signal pathways are involved in HD5_1–9_- and hBD2-mediated effects on host HDP defense.

**Figure 6:**
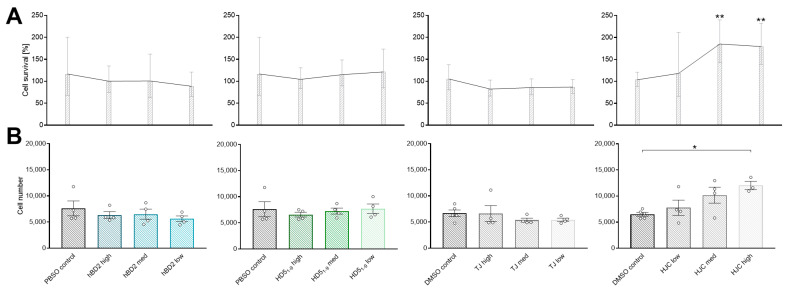
hBD2, HD5_1-9_, HJC015233, TJ-M2010-5 reveal no toxicological effects on organoids in vitro. For MTT assay, organoids were treated with hBD2 (3.6, 36, 360 ng/µL), HD5_1–9_ (3.6, 36, 360 ng/µL), HJC015233 (0.5, 5, 50 µg), or with TJ-M2010-5 (100, 50, 5 µM) for 30 h. Cell survival by quantification of fluorescence signals (*n* = 4; graphically indicated by ° for one sample each) (**A**) and total number of organoids (**B**) are shown. Data are presented as means ± SEM and were analyzed by one-way ANOVA with Dunnett’s post-test or unpaired *t*-test. Significant differences are indicated: * *p*-value < 0.05; ** *p*-value < 0.01. Abbreviations: HJC, STAT3-inhibitor HJC015233; TJ, Myd88-inhibitor TJ-M2010-5. For other abbreviations, see Figure 1.

To exclude cytotoxic effects, we first conducted an MTT assay. Analysis revealed that hBD2, HD5_1–9_, and Myd88-inhibitor TJ-M2010-5 (TJ) had no toxicological effects on cell survival or cell number (Figure 6A,B). In addition, treatment of cells with 5 µg or 50 µg STAT3-inhibitor HJC015233 (HJC) increased cell survival (*p* < 0.01), whereby 50 µg HJC even enhanced cell number (*p* < 0.05) (Figure 6A,B). However, the MTT assay data allowed us to eliminate toxicological effects. Moreover, preliminary experiments indicated that 360 ng/µL HD5_1–9_ and 36 ng/µL hBD2 were most effective, as both concentrations induced gene expression of *Defa5* (*p* < 0.01) and Reg3γ (*p* < 0.01) (Figure S6).

**Figure 7:**
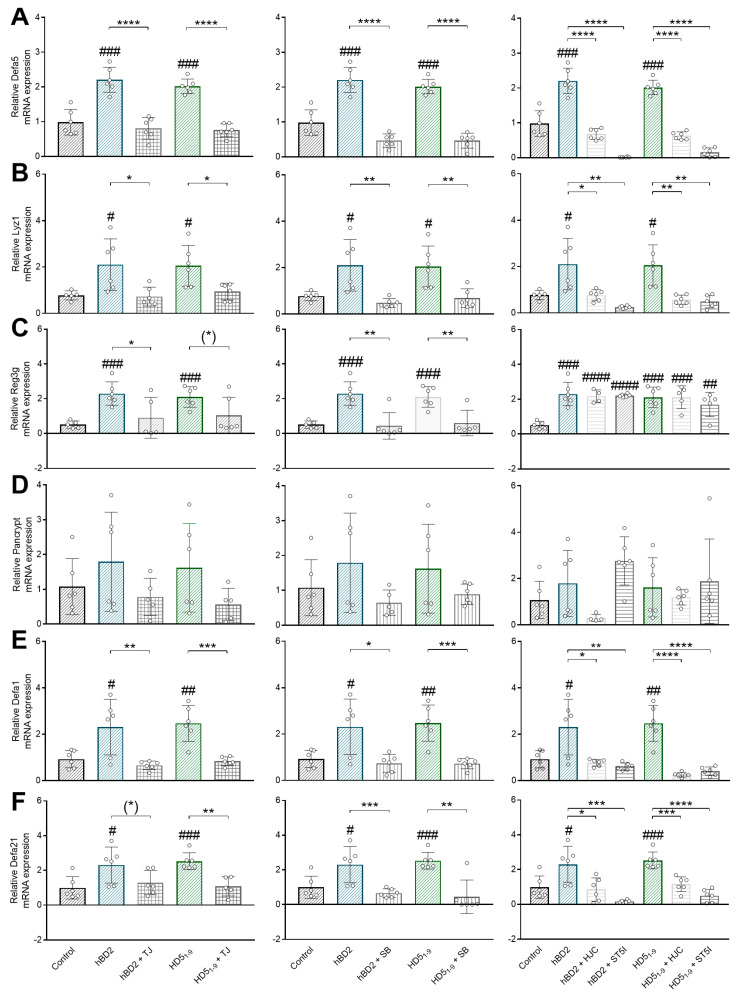
hBD2- and HD5_1-9_-induced α-Defensins in organoids in vitro through the Myd88, p38 MAPK, and Jak/STAT signaling pathways. Organoids were treated with HD5_1–9_ (1.17 µM) or hBD2 (0.027 µM) ± TJ-M2010-5 (100 µM), ± SB203 580 (5.303 mM), ± HJC015233 (5 µg), or ± STAT5-IN-1 (5 µM) for 30 h. mRNA expression levels of Defa5 (**A**), Lyz1 (**B**), Reg3g (**C**), pan-cryptidin (**D**), Defa1 (**E**), Defa21 (**F**) determined by quantitative RT-PCR derived in organoids from the small intestine of healthy C57BL/6 mice (*n* = 6; graphically indicated by ° for one sample each). Statistical analysis was performed by one-way ANOVA with Dunnett´s post-test or unpaired *t*-test. Significant differences are indicated as # *p*-value < 0.05; ## *p*-value < 0.01; ### *p*-value < 0.001; #### *p*-value < 0.0001 compared to control and as * *p*-value < 0.05; ** *p*-value < 0.01; *** *p*-value < 0.001; **** *p*-value < 0.0001 compared between groups. (*) *p*-values ranging between > 0.05 and < 0.1 were considered to indicate a trend. Abbreviations: Defa1, cryptidin 1, Defa5, α-defensin 5; Defa21, cryptidin 4; HJC, STAT3-inhibitor HJC015233; Lyz1, lysozyme; Pancrypt, pancryptidin; Reg3g, regenerating islet-derived protein 3 gamma; SB, p38 MAPK inhibitor SB203 580; ST5I, STAT5 inhibitor STAT5-IN-1; TJ, Myd88-inhibitor TJ-M2010-5.

Thirty hours stimulation of murine small intestinal organoids with 360 ng/µL HD5_1–9_ (1.17 µM) or 36 ng/µL hBD2 (0.027 µM) was found to induce gene expression of *Defa5* (*p* < 0.001), lysozyme (*p* < 0.05), and Reg3γ (*p* < 0.001) (Figure 7A–C). In contrast, neither HD5_1–9_ nor hBD2 had any effect on pan-cryptidin gene expression (Figure 7D). However, our data showed that HD5_1–9_ increased the expression of both cryptidin 1 (*p* < 0.01) and cryptidin 4 (*p* < 0.001). Similarly, hBD2 induced cryptidin 1 and 4 gene expression (*p* < 0.05) after 30 h of stimulation (Figure 7E,F).

Concurrent inhibition of the TLR4-Myd88 pathway in vitro abolished HD5_1–9_- and hBD2-induced gene expression of *Defa5* (*p* < 0.0001) and lysozyme (*p* < 0.05) (Figure 7A,B). Similarly, hBD2-mediated (*p* < 0.05) and by trend HD5_1–9_-mediated induction (*p* = 0.055) of Reg3γ were absent (Figure 7C). While pan-cryptidin expression remained unchanged (Figure 7D), blocking Myd88 resulted in a loss of hBD2- (*p* < 0.01) and HD5_1–9_- (*p* < 0.001) dependent increase of cryptidin 1 mRNA expression (Figure 7E). Consistently, cryptidin 4 expression was reduced when cells were incubated with hBD2 and TJ (trend: *p* = 0.076) or with HD5_1–9_ and TJ (*p* < 0.01) (Figure 7F).

Inhibition of p38 MAPK consistently revealed that both peptide-mediated inductions of *Defa5* (*p* < 0.0001) and lysozyme gene expression (*p* < 0.01) were absent (Figure 7A,B). Similarly, hBD2 (*p* < 0.01) and HD5_1–9_ (*p* < 0.01) failed to induce Reg3γ gene expression when p38 was inhibited (Figure 7C). Incubation with p38 inhibitor also reduced hBD2- (*p* < 0.05) and HD5_1–9_- (*p* < 0.001) dependent increment of cryptidin 1. Similarly, cryptidin 4 gene expression was reduced when organoid cells were costimulated with p38 MAPK inhibitor and hBD2 (*p* < 0.001) or HD5_1–9_ (*p* < 0.01) (Figure 7E,F).

Our organoid experiments further demonstrated that inhibition of both STAT3 and STAT5 resulted in a loss of hBD2- and HD5_1–9_-induced gene expression of *Defa5 in vitro* (*p* < 0.0001) (Figure 7A). In addition, hBD2- and HD5_1–9_-mediated induction of lysozyme gene expression was absent when STAT3 (*p* < 0.05 and *p* < 0.01, respectively) or STAT5 (*p* < 0.01) signaling was blocked (Figure 7B). In contrast, hBD2- and HD5_1–9_-dependent induction of Reg3γ mRNA expression was found to be unaffected by STAT signaling (Figure 7C). Similarly, pan-cryptidin expression was unchanged when organoid cells were incubated with STAT3 or STAT5 inhibitor (Figure 7D). Nevertheless, PCR analyses revealed that cryptidin 1 expression was neither induced by hBD2 nor HD5_1–9_ when STAT3 (*p* < 0.05 and *p* < 0.01, respectively) or STAT5 (*p* < 0.0001) were blocked (Figure 7E). Moreover, both hBD2 and HD5_1–9_ failed to enhance cryptidin 4 expression when cells were costimulated with STAT3 (*p* < 0.05 and 0.01, respectively) or STAT5 (*p* < 0.001 and 0.0001, respectively, Figure 7F).

## 3. Discussion

In the present study, we demonstrate that administration of the defensin peptides, HD5_1–9_ or hBD2, improve metabolic parameters in obese mice, such as weight gain, hepatic steatosis, and glucose metabolism. Moreover, our results strongly suggest that defensin treatments enhance intestinal barrier function, including induction of tight junction protein expression and small intestinal HDPs. Organoid cell culture experiments corroborated that HD5_1–9_- and hBD2-dependent effects on HDP expression were mediated through the Myd88, p38, and Jak/STAT signaling pathways.

The 18-week feeding of WSD±F increased body weight, mesenteric fat, and hepatic steatosis in mice, whereas CDF did not affect these parameters. These results are in contrast to findings of some previous studies from our group showing that C57BL/6J mice exhibited higher liver weight and hepatic steatosis when fed a high-fructose diet for 12 weeks [2,8]. Interestingly, we showed that only 6-week treatment with hBD2 tended to reduce mesenteric fat in g and liver weight, whereas both HD5_1–9_ and hBD2 reduced mesenteric fat cell area and fat cell size, along with liver fat accumulation in WSDF-fed mice. Concordantly, Li et al. [37] demonstrated that a 10-week treatment with the modified HD5_1–9_ fragment, termed D3, reduced body weight, especially epididymal fat weight in high-fat diet (HFD)-fed mice. Moreover, D3 reduced food intake in mice by activating uroguanylin (UGN) [37], an anorexic hormone secreted by small intestinal enterochromaffin cells, suppressing appetite via the UGN-transmembrane receptor guanylyl cyclase 2C (GUCY2C) axis [51]. Additionally, a 10-week treatment of HFD-fed mice with full-length HD5 mitigated dyslipidemia, hypercholesterolemia, and circulating fatty acid levels [34]. However, full-length HD5 failed to change body weight or epididymal white fat tissue mass [34]. In addition, hBD2 has been found to exhibit liver protective effects, as 6-week treatment of mice fed an ETOH-containing diet improved alcohol-associated liver disease (ALD), including reducing plasma alanine transaminase (ALT) activity and inducing hepatic expression of IL-17a and IL-22 [40].

In agreement with improved liver function, we were able to show that oral administration of either HD5_1–9_ or hBD2 attenuated diet-induced glucose intolerance in mice fed a WSDF, resulting in a lowering of blood glucose curves and a reduction in the AUC. Since type 2 diabetes mellitus (T2DM) and decreased HDP expression in the upper small intestinal tract and saliva was found to be associated in human studies [52,53], we were interested to quantify the effects of HD5_1–9_ or hBD2 on glucose metabolism. Although we did not observe any effects on fasting blood glucose levels, HD5_1–9_ and hBD2 improved blood glucose curves at 30 min after glucose administration in CDF- and WSDF-fed mice. Comparable findings were also reported by Larsen et al. [34], demonstrating that full-length HD5 treatment in high-fat diet (HFD)-fed mice did not change fasting blood glucose levels, but attenuated blood glucose curves at 15 and 30 min after glucose challenge. Similarly, 10-week treatment of HFD-fed mice with the modified HD5_1–9_ fragment, D3, improved glucose clearance rate comparable to those of normal chow-fed mice, indicating that the HD5_1–9_ fragment, D3, may improve obesity-induced insulin resistance [37]. Similarly, hBD2 was found to regulate diabetic wound healing in rats, as hBD2-loaded Poly-lactic-co-glycolic acid (PLGA) nanoparticles accelerated healing, which was associated with decreased Mmp9 and TNFa expression [54]. However, our results provide evidence for the first time that hBD2, comparable to HD5_1–9_, also modulates glucoregulatory capacity in WSDF-fed mice and thus could be an interesting therapeutic approach for diet-induced disturbances in glucose metabolism.

In the present study, we further demonstrated that defensin treatment improves intestinal barrier function by regulating ileal and colonic tight junction and ileal mucin expression. There is evidence that both HD5_1–9_ and hBD2 modulate gut barrier function during inflammatory processes. Specifically, the HD5-enriched diet has been shown to improve colonic ocln and ZO-1 protein expression in ethanol-induced and dextran sulfate sodium (DSS)-induced colitis in mice [33]. Consistently, Zeng et al. [55] revealed that administration of a *Lactococcus lactis* recombinant NZ9000SHD5 strain, which continuously produces mature HD5, to DSS-treated mice improved epithelial barrier integrity by reducing plasma FITC-dextran. These effects were associated with enhanced *ocln* and *ZO-1* gene expression. While our data suggested no effects of HD5_1–9_ on ileal or colonic ocln expression, we consistently found an induction of ileal *ZO-1* gene expression in WSD±F-fed mice treated with either HD5_1–9_ or hBD2. Furthermore, we demonstrated that defensin-treatment decreased the Lac/Man ratio in WSD±F-fed mice, indicating improved gut barrier function [43]. Concordantly, Han et al. [56] provided evidence that porcine β-defensin (pBD)2 decreased plasma FITC-dextran levels and enhanced *ZO-1*, *ZO-2*, *Muc1*, and *Muc2* mRNA and protein expression in the colon of DSS-treated mice [56]. Similarly, hBD2 induced gene expression of *ocln*, *cldn1*, and *ZO-1* and enhanced transepithelial electrical resistance (TEER) in Candida albicans-transfected Caco-2 cells [57]. A further in vitro model, using human keratinocytes, implied that human β-defensins modulate gene expression of several claudins by activating Ras-related C3 botulinum toxin substrate 1, atypical protein kinase C, glycogen synthase kinase-3, and phosphatidylinositol 3 kinase [58].

Consistent with our findings that showed induced Muc1 and Muc2 gene expression in the ileum of WSD±F-fed mice by peptide-treatment, 10 weeks of HD5 fragment D3 administration in HFD-fed mice enhanced colonic mucus thickness [37]. Furthermore, there is evidence that *Muc2* and defensins regulate each other. Thus, Muc2^−/−^ mice exhibited impaired colonic β-defensin 2 mRNA expression and peptide localization, as compared with wildtype littermates [59]. Complementarily, hBD2 induced Muc2 gene expression in Caco-2 and HT-29 cells [60]. Moreover, hBD2 modulates intestinal morphology, as 10-day hBD2 treatment of trinitrobenzene sulfonicacid (TNBS)-induced colitis in mice attenuated macroscopic and microscopic histology of mouse colons [38]. Collectively, these data allow us to conclude that HD5_1–9_ and hBD2 exhibit potential to attenuate intestinal barrier dysfunction.

Another important factor for intestinal barrier function are host defense peptides (HDP), which have been considered as a first line of defense in the intestinal tract [61]. Our data showed that both high fructose intake and WSD diet impaired several small intestinal HDPs, which were restored by defensin treatment. Consistently, Hodin et al. [27] showed that individuals with obesity exhibit decreased HD5 and lysozyme levels. Similarly, feeding rats with a HFD for 2 weeks resulted in bile-acid toxic effects on Paneth cells, resulting in decreased ileal lysozyme protein levels and reduced Defa5 and 6 mRNA expression [62]. While Larsen et al. [34] found no effects of a standard HFD or HD5 treatment on Defa5 gene expression in the jejunum or ileum of mice, the present study and our previous work indicate that the combination of high-fat and high-sugar diets alters ileal HDP expression [2,8], which is in line with a recent study showing how WSD promotes Paneth cell dysfunction [63]. Importantly, HD5_1–9_ and hBD2 administration partly rescued this phenotype by inducing α-defensin mRNA expression both in vitro and in vivo. However, our results demonstrated that the DEFA5 protein signal was only increased in WSD- and WSDF-fed mice receiving hBD2. These findings point toward the fact that hBD2 but not HD5_1–9_ treatment enhanced HDP protein formation.

Furthermore, we provide evidence that HD5_1–9_- but not hBD2-mediated effects on HDP gene expression in mice were associated with increased activity in the Wnt signaling pathway, as HD5_1–9_ treatment induced *Wnt3*, *5a*, *9a*, and *Tcf1* expression. The Wnt signaling pathway affects the final maturation and biological function of Paneth cells [64], and aberrant regulation of the Wnt signaling pathway has been associated with various diseases [65,66]. For example, Crohn’s disease patients showed reduced HD5 and -6 levels, which were associated with decreased *Tcf1*, *Tcf4*, and *LRP6* [19,67], along with reduced Wnt signaling molecules *Wnt1*, *-3*, and *-3a* [68]. In contrast, hyperactivation of the Wnt signaling pathway, especially by mutation in the adenomatous polyposis coli (APC) gene, has been associated with colorectal carcinogenesis [69]. However, there is evidence that overexpression of *Defa5* reduced tumorigenesis in mice by directly binding phosphoinositide 3-kinases (PI3K) subunits [70].

By using an organoid cell-culture model, we demonstrated that HD5_1–9_ and hBD2 induce HDPs through the Myd88 signaling pathway in vitro. Recent studies revealed that bacterial-mediated regulation of HDP expression occurs in a TLR/Myd88-dependent manner [71,72]. Thus, microbiota-free, TLR- or Myd88-deficient mice exhibited decreased Defa5 mRNA expression, which was recovered by treating with *Lactobacillus lactis* or TLR agonists [73]. Moreover, activation of TLR2 and -4 by LPS and peptidoglycan-stimulated α-defensin-2 promoter activation in Caco-2, T84, and SW480 cells [74]. Consistent with our data, Funderburg et al. [49] demonstrated that hBD3 induces expression of costimulating molecules such as CD80, 86 and 40 on monocytes by interactions with TLR-1 and -2, resulting in enhanced Myd88 signaling. These results suggest that TLR signaling is not restricted to microbial signals but also can be activated by HDPs.

Furthermore, we demonstrated that HD5_1–9_ and hBD2 effects were dependent on the MAPK/p38 signaling pathway. In addition to data showing that hBD2 and hBD3 levels are regulated by probiotics via p38 and NF_K_B activity [75], there is evidence that defensins also modulate different immunology processes through p38 signaling. In vitro data revealed that hBD1-4 induced the secretion of angiogenic factor ANG of human dermal fibroblasts by increasing phosphorylation of p38 and c-Jun N-terminal kinases (JNK) [50]. Complementarily, hBD-2, -3, and -4, and LL-37 increased IL-18 mRNA expression in human keratinocytes, which has been associated with anti-inflammatory effects [76]. Similarly, HD6 has been characterized to improve the outcome of colorectal cancer in vivo, whereby in vitro data indicated that HD6 overexpression reduced cell proliferation and serpine-1 expression, by suppressing nuclear translocation of p38 and JNK [77].

The Jak/STAT signaling pathway mediates immune regulatory processes, such as cytokine expression and inflammatory response [78]. We demonstrated that defensin-mediated effects on HDP gene expression were dependent on STAT3 and STAT5 signaling. Concordantly, it has been reported that hBD3 enhanced phosphorylation of Jak2 and STAT3 in human dermal fibroblasts, resulting in increased production of angiogenic growth factors, which has been associated with improved wound healing [48]. Moreover, salidroside, a biological active component with anti-inflammatory properties, regulated *Defa5* and *-6* expression in intestinal epithelial 6 cells (IEC-6) from rats by regulating STAT3 activity [79]. Similarly, the absence of STAT5 in transgenic mice has been found to reduce Paneth cells and to increase predisposition of mice for *Clostridium difficile* infection [63]. Moreover, STAT5^−/−^ mice exhibited deregulations in intestinal epithelial stem cells markers and an impaired crypt regeneration after radiation-induced mucositis [80], suggesting that STAT5 could be also required for Paneth cell differentiation and regulation of intestinal HDPs.

A possible limitation in the present study is that we did not analyze intestinal stability of HD5_1–9_ and hBD2 in mice. There is evidence that peptides generally display a low bioavailability due to proteolytic degradation [81]. Thus, the reduced form of HD5 full peptide HD5_red_ was found to be degraded into different fragments such as HD5_1–9_ by intestinal proteases [26]. However, fragment formation has been associated with enhanced antimicrobial activity of HD5, wherein especially the resulting fragment HD5_1–9_ exhibited comparable antimicrobial activity to the full HD5 peptide [26]. Further, evidence was obtained for the human neutrophil α-defensin 4 (HNP4) fragment _1–11_ that neither proteolysis nor reduction further degraded the fragment nor affected antimicrobial activity [82]. A comparable mechanism was also shown for β-defensins. Wendler et al. [83] demonstrated that reduced β-defensin 1 (hBD1_red_) was proteolytically degraded into different fragments, which exhibited increased antimicrobial activity and inhibited growth of *Bifidobacterium breve*. Nevertheless, oral administration of HD5 has been shown to provide therapeutic effects, as treatment with modified HD5_1–9_ fragment D3 regulated appetite in obese mice [37], and oral administration of HD5 improved glucose tolerance [34], gut barrier function, and microbiota composition in mice after radiation-induced injury [84]. Similarly, intranasal treatment of hBD2 improved experimental asthma [85] and oral hBD2 administration ameliorated alcohol-associated liver disease in mice [40]. Therefore, the results of the present study and previous work point toward the fact that the HD5_1–9_ fragment and hBD2 full peptide provide pharmacokinetically effects in vivo.

In summary, the present study strongly suggests that both HD5_1–9_- and hBD2-treatment ameliorate metabolic parameters such as hepatic steatosis and glucose metabolism but also improve intestinal barrier function, especially tight junction protein expression and small intestinal HDP defense, in mice fed a low-fiber WSD enriched with fructose. Moreover, in vitro data indicated that peptide-mediated effects on HDP expression were dependent on the Myd88, p38, and Jak/STAT signaling pathways. Our results suggest that HDPs could be an interesting therapeutic approach for obesity and obesity-related comorbidities, but it is important to mention that production and extraction of whole HDP structures are difficult and expensive. The use of defensin fragments such as HD5_1–9_ represents an opportunity to produce lower-cost bioactive compounds more easily. Since the present and other animal studies did not observe side effects of HD5 or hBD2 treatment, it is conceivable that HDPs could be a useful therapeutic tool for clinical practice. There are still studies necessary, evaluating whether defensin treatment has comparable effects on metabolic parameters in humans and which doses are required for pharmacokinetic effects.

## 4. Materials and Methods

### 4.1. Experimental Setup, Animals, and Diets

Experimental setup was carried out with 84 male C57BL/6J mice. Three 6–8-week-old mice per cage were cohoused in a specific pathogen-free (SPF) barrier facility with a controlled 12-h light/dark cycle, accredited by the Association for Assessment and Accreditation for Laboratory Animal Care International. The local Animal Care and Use Committee approved all experiments (Regional Council Stuttgart 362/20 EM). Mice were randomly divided into four dietary groups (*n* = 21 per diet), receiving either a compositionally defined control diet (CD, Ssniff^®^ CD88137, Soest, Germany) or a Western-style diet (WSD, Ssniff^®^ TD88137, Soest, Germany) for 18 weeks. Autoclaved tap water either with or without supplementation of D-(–) fructose 30% weight/weight (F, 5.09 KJ/g (1.22 kcal/g), >99.5% purity, Carl Roth, Karlsruhe, Germany) was offered ad libitum to mice. The detailed compositions of administered diets are shown in Table 2. Food and fluid intake together with body weight were assessed weekly throughout intervention. After 12 weeks, gut barrier tests together with an oral glucose tolerance test (oGTT) were performed, and animals were randomly divided into three intervention groups (*n* = 7 per intervention and diet). Following these measurements, mice were daily gavaged in the morning with either the recently discovered and biologically active HD5_1–9_ (1.2 mg/kg), or full peptide hBD2 (1.2 mg/kg), or 0.01% BSA in PBS as control for six weeks. To determine gavage volume (1.2 mg/kg bw), mice were weighed three times per week. At week 20, intestinal barrier tests and oGTT were repeated. Afterward, mice were anesthetized and blood, liver, mesenteric fat tissue, and gut tissue specimens were collected and stored, as previously described [8].

### 4.2. Synthesis and Purification of Peptide Fragment HD5_1–9_ and Full Peptide hBD2

HD5_1–9_ was chemically synthesized by EMC Microcollections GmbH (Tübingen, Germany) and purified by precipitation. The structure of the HD5_1–9_ was confirmed by RP-HPLC-ESI-MS (purity ≥90%) and HD5_1–9_ was dissolved in PBS. Recombinant hBD2 was expressed in *E. coli*, as described in the patent (WO2010/007166), and provided by Defensin Therapeutics. Proper folding and disulfide bridge topology was verified as previously described [38]. Recombinant hBD2 (endotoxin levels <0.05 EU/mL) was kept in its natural tertiary structure (purity ≥96%) and was dissolved in PBS. 

### 4.3. Measurement of Intestinal Permeability

In week 13 and week 20, mice were fasted for 6 h (h) before the intestinal permeability tests were performed by using the lactulose (Carl Roth, Karlsruhe, Germany) to mannitol (Sigma-Aldrich, St. Louis, MO, USA) ratio as measured in urine. The procedure was performed as previously described [86].

### 4.4. Oral Glucose Tolerance Test (oGTT)

To evaluate glucose metabolism, an oral glucose tolerance test (oGTT) was carried out at week 13 and week 20. After fasting mice for 4 h, fasting blood glucose was determined using tail-vein blood; 20% glucose solution (3 mg glucose/g bw; Sigma-Aldrich, Steinheim, Germany) was administered by gavage and blood glucose levels were measured repeatedly after 15, 30, 45, and 60 min. From these measurements, blood glucose curves were plotted and the area under the curve (AUC) was calculated using the following equation:

$$AUC=\left(0.2C_{0\;min}\right)+\left(0.4\times C_{15\;min}\right)+\left(0.6\times C_{30\;min}\right)+\left(0.8\times C_{45\;min}\right)+\left(1\times C_{60\;min}\right)$$

### 4.5. Histological and Immunohistochemical Quantification of Liver, Gut, and Fat Tissue

Hematoxylin/eosin (HE) staining was performed in 5 µm thick paraffin sections of gut, liver, and mesenteric fat tissue, as previously described [87]. For immunohistochemical quantification of α-Defensin 5 (DEFA5), 3 µm thick paraffin sections of ileum were prepared, followed by DEFA5-staining, as reported in detail elsewhere [86]. Optical density measurements by a fluorescence microscope (Axiovert 200 M, AxioVision 4.8.2 SP3; Carl Zeiss AG, Aalen, Germany) were performed. The total area of the fluorescence signal (%) in a fixed frame (width: 200.273 µm; length: 333.78 µm) was determined using an automatic measurement program. For each slide, 5 images were created and evaluated.

### 4.6. Organoid Cell Culture

#### 4.6.1. Isolation and Purification

For intestinal organoids, crypts from C57BL/6J mice were used. Mice were anesthetized using CO_2_ and crypts were isolated by crypt isolation buffer (CIB, PBSO containing 0.5 M EDTA). A total of 500 crypts were plated with 25 µL Matrigel (Corning B.v., Amsterdam, the Netherlands). After polymerization at 37 °C, 300 µL of crypt culture medium (CCM) was added and the resulting organoids were cultured and split as described [86].

#### 4.6.2. Assessment of Cell Viability by Measuring MTT Reduction

To evaluate the effects of HD5_1–9_, hBD2, STAT3-inhibitor HJC015233, and Myd88-inhibitor TJ-M2010-5 on organoid cell viability, a MTT reduction assay was performed, as previously reported [86]. Concentrations of STAT5 inhibitor STAT5-IN-1 and p38 inhibitor SB203 580 were already identified in previous work [86]. Organoids were incubated with HD5_1–9_ (3.6, 36, 360 ng/µL, solved in BSA/PBSO), or hBD2 (3.6, 36, 360 ng/µL, solved in BSA/PBSO), or HJC015233 (0.5, 5, 50 µg, solved in DMSO; Selleckchem, Houston, TX, USA), or TJ-M2010-5 (100, 50, 5 µM solved in DMSO; MedChemExpress, Sollentuna, Sweden), or with a corresponding amount of BSA/PBSO or DMSO as control for 30 h.

#### 4.6.3. Treatment of Organoids

Organoids were treated with HD5_1–9_ (1.17 µM, solved in BSA/PBSO), or hBD2 (0.027 µM, solved in BSA/PBSO), or corresponding amount of BSA/PBSO as control for 30 h. Furthermore, cells were incubated with HD5_1–9_ or hBD2 and STAT3-inhibitor HJC015233 (5 µg, solved in DMSO; Selleckchem, Houston, TX, USA), or STAT5-inhibitor STAT5-IN-1 (5 µM, solved in DMSO; Selleckchem, Planegg, Germany), or p38 inhibitor SB203 580 (5.303 mM, solved in DMSO; Sigma-Aldrich, Darmstadt, Germany), or Myd88 inhibitor TJ-M2010-5 (100 µM, solved in DMSO; MedChemExpress, Sollentuna, Sweden) for 30 h. A corresponding amount of DMSO was used as a control.

### 4.7. RNA-Extraction and Quantification of Gene Expression by Real-Time PCR in Mouse Tissue and Organoid Cells 

Extraction of total RNA was performed by using peqGOLD TriFast system (PEQLAB, Erlangen, Germany) for mouse tissue or ExtractME Total RNA Kit (blirt S.A., Hilden, Germany) for organoid cells. For cDNA synthesis, a reverse transcription system kit with random primers was used and real-time (RT)-PCR was performed by using oligonucleotide primers listed in Supplemental Table S1, as previously described [8]. Absolute gene expression was calculated by normalizing copy numbers to housekeeping gene β-actin and relative gene expression was quantified by using the ΔΔ-Ct method.

### 4.8. Statistical Analysis

GraphPad Prism software 7.0 (GraphPad Software Inc., La Jolla, CA, USA) was used for all statistical analysis. The Kolmogorov–Smirnov test was performed to examine data for normal distribution. For nonparametric data, the Kruskal–Wallis test followed by Dunn’s test or one-way-ANOVA and Dunnett´s post-test were used. Depending on Gaussian distribution, an unpaired *t*-test or Mann–Whitney test was performed to evaluate differences between two groups. To evaluate effects of time and intervention, a two-way ANOVA with Tukey’s multiple comparisons test was performed. *p*-values of *p* < 0.05 were considered as statistically significant and *p*-values ranging between > 0.05 and < 0.1 were considered to indicate a trend.

## 5. Patents

Aesculus Bio (AB) ApS holds a matter-of-substance patent related to HD5_1–9_ for pharmaceutical use. Defensin Therapeutics (DT) ApS holds patents for pharmaceutical use of hBD2.

## Figures and Tables

**Table 1:** Regulators of antimicrobial peptides in ileal tissue.

	WSD	WSD-HD5	WSD-hBD2	WSDF	WSDF-HD5	WSDF-hBD2
**Wnt 3**	2.109 ± 0.702	7.215 ± 5.367	0.843 ± 0.262	1.057 ± 0.169	**19.43 ± 7.718 ##**	1.121 ± 0.222
**Wnt 5a**	2.004 ± 0.346	4.6 ± 1.864	1.46 ± 0.181	1.082 ± 0.1411	**3.172 ± 0.94 #**	0.844 ± 0.198
**Wnt 9a**	1.444 ± 0.498	12.85 ± 7.796	1.204 ± 0.1965	0.442 ± 0.0771	**16.93 ± 7.879 #**	0.484 ± 0.0699
**LRP6**	1.389 ± 0.255	1.295 ± 0.228	0.859 ± 0.129	0.7449 ± 0.084	0.91 ± 0.175	0.709 ± 0.061
**Tcf1**	1.297 ± 0.1452	0.891 ± 0.251	0.886 ± 0.195	0.639 ± 0.07	**6.451 ± 2.489 ##**	0.647 ± 0.1224
**Tcf4**	0.879 ± 0.19	0.912 ± 0.114	1.248 ± 0.121	1.09 ± 0.175	1.096 ± 0.167	1.254 ± 0.211
**Mmp7**	2.428 ± 1.176	1.131 ± 0.229	2.382 ± 1.766	2.54 ± 0.989	2.973 ± 1.528	1.286 ± 0.385

HD5_1-9_ modulated activity of the ileal Wnt signaling pathway. mRNA expression of Wnt3, Wnt5a, Wnt9a, LRP6, Tcf1, Tcf4, and Mmp7 in the ileum was determined by quantitative RT-PCR. Data are shown as means +/− standard error of the mean (*n* = 6–7). Statistical analysis was performed by the Kruskal–Wallis test with Dunn’s test or by one-way ANOVA with Dunnett´s post-test. Statistics: # indicates differences relative to WSDF. # *p*-value < 0.05; ## *p*-value < 0.01. Abbreviations: ATF, activating transcription factor 4; BiP, chaperone protein-binding protein; CD, control diet; F, fructose; HD5, human α-defensin 5; hBD2, human β-defensin 2; LRP, low-density-lipoprotein receptor related protein 6; Tcf, T cell-specific transcription factor; Wnt, wingless and Int; WSD, Western-style diet.

**Table 2:** Nutrient formulation of experimental diets fed to male C57BL/6J mice for 18 weeks.

	CD Ssniff ^®^ E15000-347	WSD Ssniff^®^ E15721-347
Diet specification	CD	WSD
ME (MJ/kg)	15.7 (3.7 kcal/g)	19.1 (4.575 kcal/g)
Carbohydrates (kJ%)	66	43
Mono- and Disaccharides (g/kg)	111	343
Protein (kJ%)	23	15
Fat from soybean oil (kJ%)	13	-
Fat from butter fat (kJ%)	-	42
Cholesterol (mg/kg)	-	2.07
Minerals (g/kg)	60	378
Sodium (g/kg)	1.6	2.4
Starch (g/kg)	476	144
Crude ash (g/kg)	54	42
Crude fiber (g/kg)	50	50
Crude protein (g/kg)	211	173
Crude fat (g/kg)	41	211

Abbreviations: ME, metabolizable energy; CD, control diet; WSD, Western-style diet.

## Data Availability

The data presented in this study are available upon justified request to the corresponding author.

## Supplementary Materials

The following supporting information can be downloaded at: https://www.mdpi.com/article/10.3390/ijms241813878/s1.
