# Supplementary material for: Human α-Defensin 51–9 and Human β-Defensin 2 Improve Metabolic Parameters and Gut Barrier Function in Mice Fed a Western-Style Diet
Source: Int J Mol Sci. 2023 Sep 9;24(18):13878. doi: 10.3390/ijms241813878 (PMC10531064; doi:10.3390/ijms241813878)
Supplement: Supplementary file 1 [file ijms-24-13878-s001.zip › ijms-2586730-supplementary.pdf]

Supplements

Table S1. Primers are used in quantitative real-time PCR.

Primer	Forward (5′-3′)	Reverse (5′-3′)
Actb	GCTGAGAGGGAAATCGTGCGTG	CCAGGGAGGAAGAGGATGCGG
Pan-cryptidin	AAGAGACTAAAACTGAGGAGCAGC	GGTGATCATCAGACCCCAGCATCAGT
Defa1	TCAAGAGGCTGCAAAGGAAGAGAAC	TGGTCTCCATGTTCAGCGACAGC
Defa5	TCAAAAAAGCTGATATGCTATTG	AGCTGCAGCAGAATACGAAAG
Defa21	CCAGGGGAAGATGACCAGGCT	TGCAGCGACGATTTCTACAAAGGC
Lyz1	GCCAAGGTCTACAATCGTTGTGAGTTG	CAGTCAGCCAGCTTGACACCACG
Reg3g	TTCCTGTCCTCCATGATCAAAA	CATCCACCTCTGTTGGGTTCA
Mmp7	TTCAAGAGGGTTAGTTGGGGGACTG	CCGCCTCTACGAGTGAAACTGTT
Wnt3	TGGAACTGTACCACCATAGATGAC	GTAGCGGAGCCGACCACA
Wnt5a	ATGGTGTGTCTGGCTCCTG	CAGTGGCTTCATTGGTAGTGCT
Wnt9a	GCAGGACTTTCTCAAGGACA	CCCTGCCAAAGACAGAAGTA
LRP6	TGGTGGCTTGTGGTGCTG	CAATGGCGATGCGAACTG
Tcf1	CCAGGACTCACCCTCGTA	TTCTGTGCCTAGCAACCAA
Tcf4	CTGGTCTGCACGGGATAA	TGTGACTTGGCGTCTTGG
Muc1	TCCTTCTGAGAGCCACCACT	GGTGACCACTTCTGCCAACT
Muc2	GATGGCACCTACCTCGTTGT	GTCCTGGCACTTGTTGGAAT
Cldn2	GTCATCGCCCATCAGAAGAT	ACTGTTGGACAGGGAACCAG
Cldn5	GCTCTCAGAGTCCGTTGACC	CTGCCCTTTCAGGTTAGCAG
Cldn7	GCGACAACATCATCACAGCC	CCTTGGAGGAATTGGACTTGG
Ocln	ACTCCTCCAATGGACAAGTG	CCCCACCTGTCGTGTAGTCT
ZO-1	CCACCTCTGTCCAGCTCTTC	CACCGGAGTGATGGTTTTCT

Abbreviation: Actb, β-Actin; pan-cryptidin, pancryptidin; Defa1, cryptidin 1; Defa5, α-defensin 5; Defa21, cryptidin 4; Lyz1, lysozyme; Reg3g, regenerating islet-derived protein 3 gamma; Mmp7, matrix metalloproteinase-7; Wnt, wingless and Int; LRP6, low-density-lipoprotein receptor related protein 6; Tcf, T cell-specific transcription factor; Muc, Mucin; Cldn, Claudin; Ocln, Occludin, ZO-1, zonula occludens 1.

Figure S1:

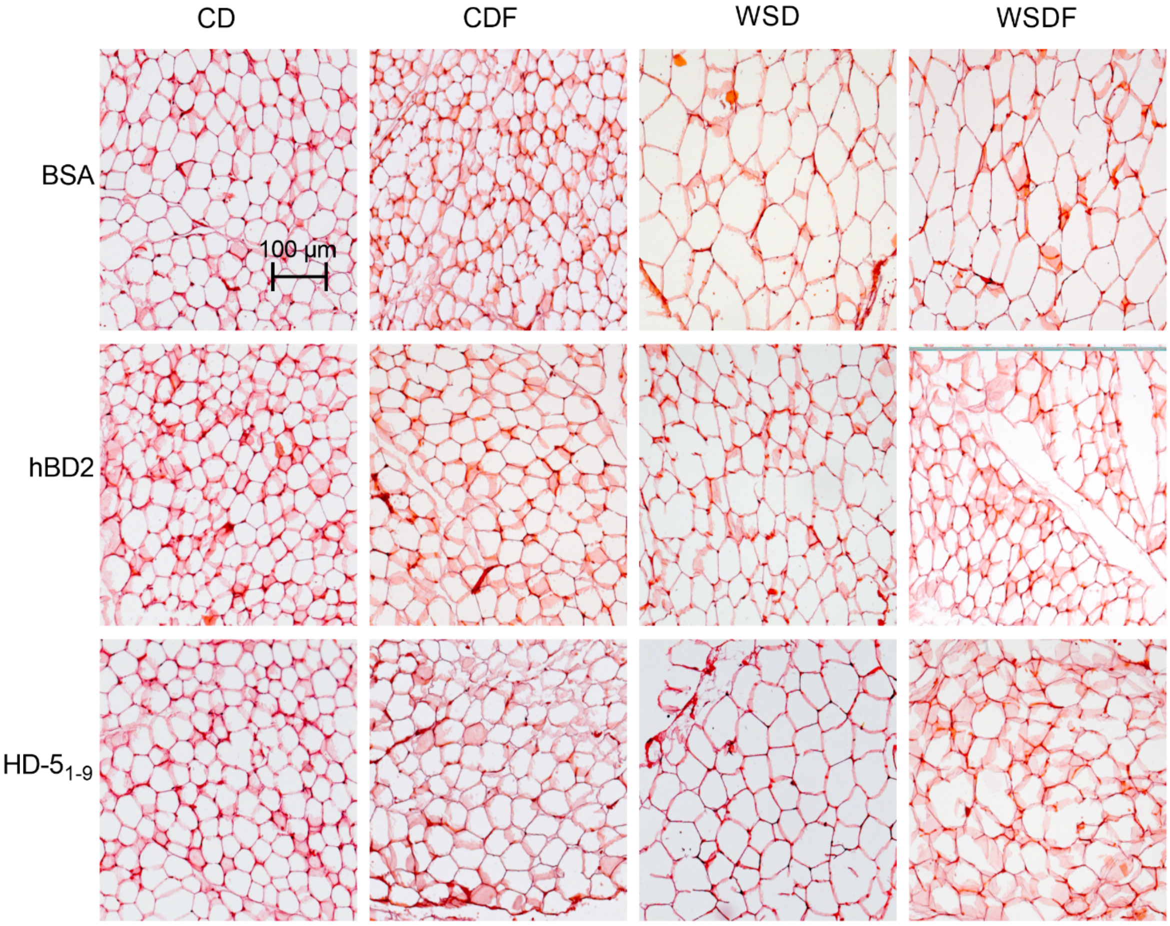

Figure S1. HD5$_{1-9}$ and hBD2 reduced mesenteric fat cell area and fat cell size in mice fed a WSD±F. Representative images of HE staining in mesenterial fat tissue. Scale bar: 100 µm (n = 6-7). Abbreviations: CD, control diet; F, fructose; HD5, human α-defensin 5; hBD2, human β-defensin 2; WSD, Western-Style diet.

Figure S2

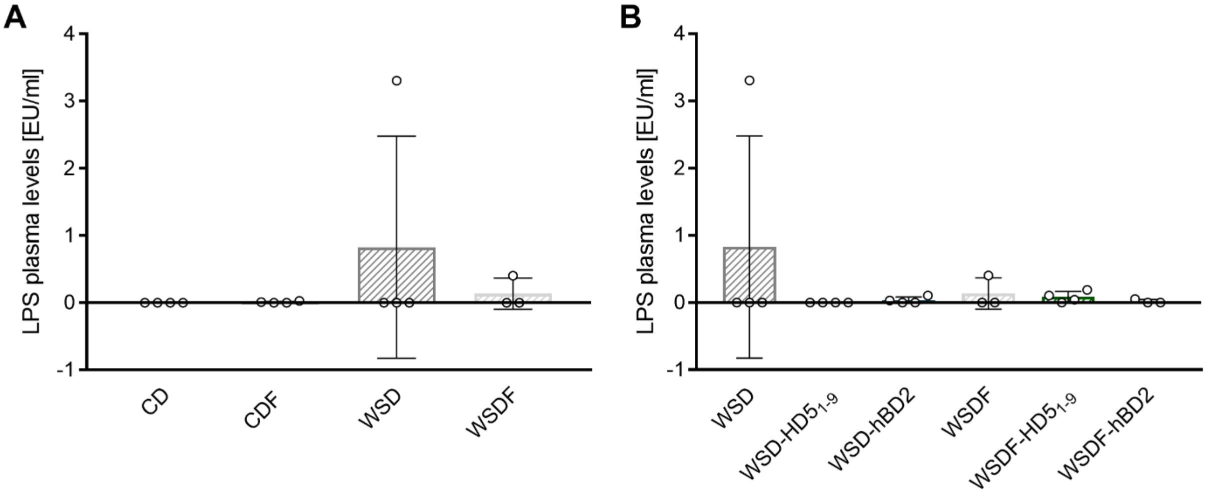

Figure S2. Endotoxemia in the portal vein plasma was unaffected by diets and defensin treatments. LPS plasma levels in EU/ml in the diet groups (A), LPS plasma levels in EU/ml in the intervention groups (B). Data are presented as means +/- standard error of the mean (SEM) (n = 6-7). Statistical analysis was performed by Kruskal-Wallis-test with Dunn's test. Abbreviations: CD, control diet; F, fructose; EU, endotoxin units; HD5, human α-defensin 5; hBD2, human β-defensin 2; WSD, Western-Style diet.

Figure S3:

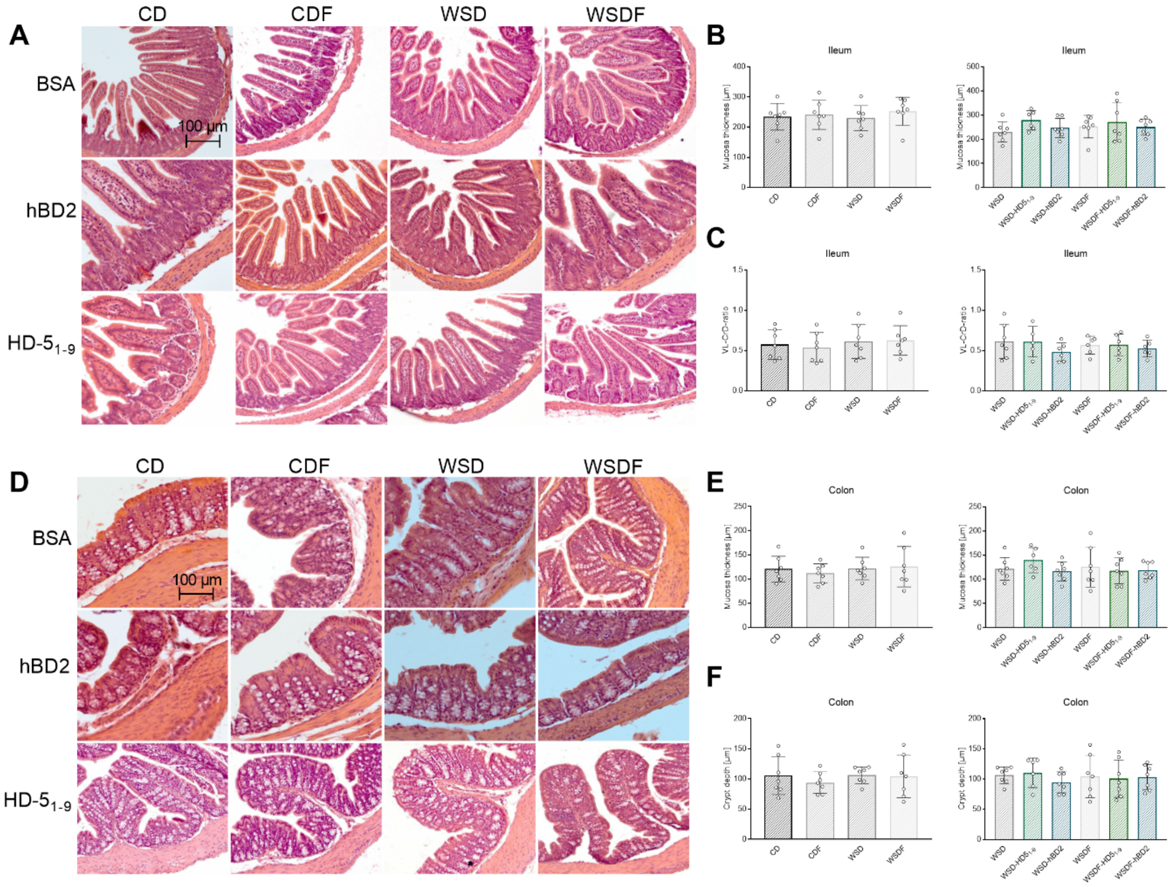

Figure S3. Intestinal crypt depth and villus length remained unchanged. Representative images of HE staining in ileal and colonic tissue. Scale bar: 100 µm (A, D). Quantification of mucosa thickness (B), ratio of villus length to crypt depth (C) in the ileum, as well as mucosa thickness (E) and depth of the crypts (F) in the colon (G). Data are presented as means +/- standard error of the mean (n = 6-7). Statistical analysis was performed by one-way ANOVA with Dunnett´s post-test. Abbreviations: CD, control diet; F, fructose; HD5, human α-defensin 5; hBD2, human β-defensin 2; VL-CrD-ratio, ratio of villus length to crypt depth; WSD, Western-Style diet.

Figure S4:

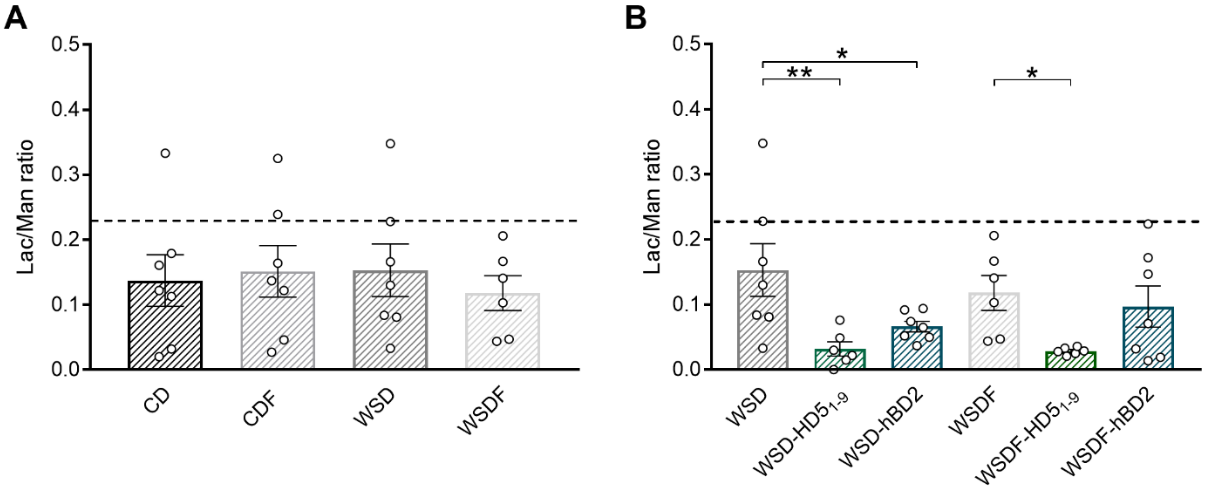

Figure S4. Diets did not affect intestinal permeability, but HD5 1-9 and hBD2 treatment improved gut barrier integrity. Intestinal permeability was determined by Lac/Man ratio in 18 h urine samples (n=6-7) in the diet groups (A) and the intervention groups (B). Data are presented as means +/− standard error of the mean (n = 6-7). Statistical analysis was performed by one-way ANOVA with Dunnett´s post-test. Significant differences are indicated: * p-value < 0.05, ** p-value < 0.01. Abbreviations: CD, control diet; F, fructose; HD5, human α-defensin 5; hBD2, human β-defensin 2; Lac; lactulose; Man, mannitol; WSD, Western-Style diet.

Figure S5:

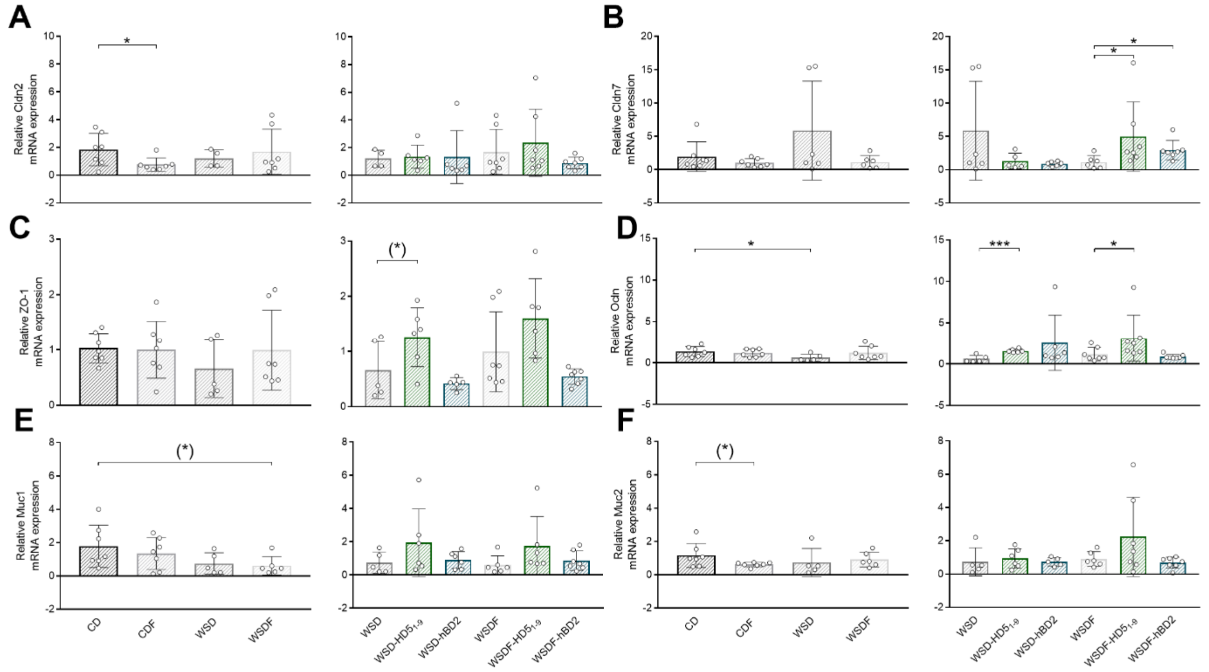

Figure S5. Antimicrobial peptide treatment regulates intestinal barrier function in the colon. mRNA expression levels of cldn2 (A), cldn7 (B), ZO-1 (C), ocln (D), Muc1 (E), Muc2 (F) in colon of mice after 18-week feeding period are shown. Data are presented as means +/− standard error of the mean (n = 6-7). Statistical analysis was performed by Kruskal-Wallis-test with Dunn's test. Significant differences are indicated: * *p*-value < 0.05; *** *p*-value < 0.001. *p*-values ranged between > 0.05 and < 0.1 were considered as trend. Abbreviations: cldn, claudin; Muc, mucin; ocln, occludin; ZO-1, zonula occludens 1; for other abbreviations, see Figure 1.

Figure S6:

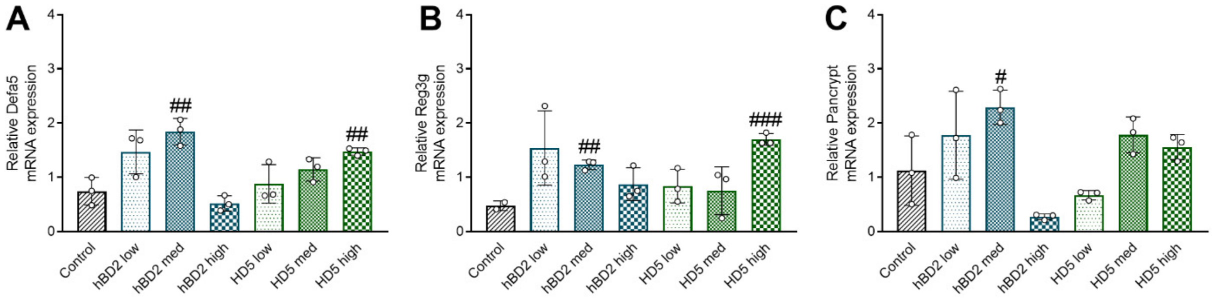

Figure S6. To induce α-Defensins in organoids *in vitro*, 36 ng/µL of hBD2 and 360 ng/µL of $HD5_{1-9}$ were most effective. Organoids were treated with $HD5_{1-9}$ (3.6 ng/µL, 36 ng/µL, 360 ng/µL) or hBD2 (3.6 ng/µL, 36 ng/µL, 360 ng/µL) for 30h. mRNA expression levels of Defa5 (A), Reg3g (B), and pan-cryptidin (C) determined by quantitative RT-PCR derived in organoids from the small intestine of healthy C57BL/6 mice (n=3). Statistical analysis was performed by unpaired t-test. Significant differences are indicated as # p-value < 0.05; ## p-value < 0.01; ### p-value < 0.001 compared to control. Abbreviations: Defa5, α-defensin 5; Pancrypt, pancryptidin; Reg3g, regenerating islet-derived protein 3 gamma; for other abbreviations, see Figure 1.
